# Insightful Advancement and Opportunities for Microbial Bioplastic Production

**DOI:** 10.3389/fmicb.2021.674864

**Published:** 2022-01-04

**Authors:** Kanchan Samadhiya, Rimjhim Sangtani, Regina Nogueira, Kiran Bala

**Affiliations:** ^1^Department of Biosciences and Biomedical Engineering, Indian Institute of Technology, Indore, India; ^2^Institute for Sanitary Engineering and Waste Management, Leibniz Universitaet Hannover, Hanover, Germany

**Keywords:** microbial bioplastic, polyhydroxyalkanoates production, mixed microbial cultures, algae-bacteria consortia, algae

## Abstract

Impetuous urbanization and population growth are driving increased demand for plastics to formulate impeccable industrial and biomedical commodities. The everlasting nature and excruciating waste management of petroleum-based plastics have catered to numerous challenges for the environment. However, just implementing various end-of-life management techniques for assimilation and recycling plastics is not a comprehensive remedy; instead, the extensive reliance on finite resources needs to be reduced for sustainable production and plastic product utilization. Microorganisms, such as bacteria and algae, are explored substantially for their bioplastic production repertoire, thus replacing fossil-based plastics sooner or later. Nevertheless, the utilization of pure microbial cultures has led to various operational and economical complications, opening the ventures for the usage of mixed microbial cultures (MMCs) consisting of bacteria and algae for sustainable production of bioplastic. The current review is primarily focuses on elaborating the bioplastic production capabilities of different bacterial and algal strains, followed by discussing the quintessence of MMCs. The present state-of-the-art of bioplastic, different types of bacterial bioplastic, microalgal biocomposites, operational factors influencing the quality and quantity of bioplastic precursors, embracing the potential of bacteria-algae consortia, and the current global status quo of bioplastic production has been summarized extensively.

## Bioplastics “State-of-the-Art”

The cataclysmic repercussions of disposable plastics on the environment have been a great deal of concern in today’s scenario. The perennial nature of petroleum-based plastics instigates a conspicuous challenge for the ecological community in terms of their eradication and recovery. The recovery and recycling of plastic waste immensely depend on the method of disposal of plastic waste. The mixed disposal of plastic waste makes its recovery difficult and less energy efficient (Devasahayam et al., 2019). Several environmental and health-related issues are culminating every day due to extensive plastic production and usage, followed by infelicitous end-of-life management (Adane and Muleta, 2011). Apart from the convenient and cheap plastic products, single-use plastics (SUPs) have been rapidly making a mark within our busy and evolving society. Not only plastics are polluting our environment but also when exposed to various environmental factors like ultraviolet rays, temperature, etc., they break down into microplastic (MP), and this phenomenon can occur not only to disposed plastic but also the ones still in use. The chemicals that are used in manufacturing of SUPs (Phthalic acid esters) can transfer to the material contained within and travel to our food chain (Giacovelli et al., 2018; Chen et al., 2020).

The hike in plastic production has considerably surpassed any other human-made objects in the past 65 years. The complex nature of petroleum-based plastic leads to the deterioration of the environment at the expense of economic benefits. A seismic shift in the environmental policies effectuated by the overwhelming and escalating plastic pollution has redirected the prevailing maneuvers toward the advancement of native low-carbon, circular economies, with strict landfill waste regulation, systematic recycling and recovery methodologies, and compulsory usage of bioplastics for the production of innumerable commodities (Kaeb et al., 2016; Elmarasi, 2017; Godfrey, 2019). In order to reduce energy requirements and carbon footprint, researchers all over the world envision the replacement of fossil-based plastic with carbon-neutral bio-based plastic for the betterment of the environment.

### Know-How of Microbial Bioplastics

Bioplastics are biodegradable polymeric compounds composed of covalently bonded monomeric units. They are primarily derived from different biological sources, such as plants, bacteria, microalgae, and photosynthetic bacteria (Xia et al., 2021). Major components of the cells which include protein, polysaccharides, lipids, amino acids (Gonzalez-Gutierrez et al., 2010), and polyhydroxyalkanoates have been discovered as the key constituents of bio-based biodegradable plastics. Their accumulation is triggered in the microbial cell when subjected to varying physico-chemical perturbations (Samantaray and Mallick, 2015). Aliphatic polyesters, such as thermoplastic starch, polyhydroxyalkanoates, polybutylene succinate, and polylactic acid, have been acknowledged as the building block of the bioplastic products currently present in the market (Debuissy et al., 2018). Various bacterial strains including *Bacillus* sp., *Azotobacter* sp., *Alcaligenes* sp., *Pseudomonas* sp., methylotrophs, and *Cupriavidus nectar* as well as algal strains *Spirulina platensis*, *Nostoc muscorum*, *Synechococcus* sp., etc., have been explored extensively for the production of biopolymers (Lee, 1996; Steinbüchel and Füchtenbusch, 1998; Nishioka et al., 2001; Salehizadeh and Van Loosdrecht, 2004; Mallick et al., 2007; Toh et al., 2008; Babu et al., 2013; Hoarau et al., 2018).

Taking into consideration the prevailing plastic pollution and augmenting demand for plastic products, emphasizes on employing the biorefinery framework for cultivation of microalgae. Furthermore, while focusing on the biorefinery approach, it encompasses maneuvering the sustainable conversion of cell biomass into a spectrum of bio-based marketable products (bioplastics, biogas, bioethanol, pigments, protein, carbohydrates, and biofuels) to make microalgal biomass an economic-friendly and feasible feedstock for replacing the conventional plastic (Trivedi et al., 2015; Das et al., 2018; Chandra et al., 2019). Life cycle assessment (LCA) of various fossil-based and bio-based plastics has postulated that the usage and production of the later are advantageous over the former, primarily in terms of redemption of fossil reservoirs and attenuation of carbon emissions (Harding et al., 2007; Broeren et al., 2017). The bioplastics extracted from biomass feedstock and agricultural raw material tend to mimic various physical and mechanical properties of traditional plastics, and its substantial degradability by microbes has also been inferred by the researchers (Wu, 2012; Kim et al., 2020; Rocha et al., 2020). In the current view, agricultural feedstocks, such as wheat, sugar, potatoes, corn, rice, and soya, are subjected to fermentation to produce different types of bioplastics (Kadouri et al., 2005). However, continuing this practice might adversely influence the provision of food all over the world. Thus, the need for cost-effective bioplastic raw material, including microbial biomass, emerges out to be an effective solution for sustainable and feasible production of biodegradable or non-biodegradable bioplastic (Solaiman et al., 2006; Pakalapati et al., 2018).

However, the concurrent and critical scrutinization of bioplastic had suggested significant obligations in its development and commercialization, including the higher cost of bio-based substrates, microbe cultivation methodologies, and downstream processing. Perhaps, rethinking and considering the synergized cost of bioplastic production and waste management, while focusing mainly on various inexpensive biological substrates required for improved microbial growth, such as various effluents from industries, byproducts of industries like whey or crude glycerol or simply municipal waste (Amaro et al., 2019; Tamang et al., 2019; Wen et al., 2020) might give us a new perspective for economic and environmental evaluation of bioplastic regulatory framework comprising of its production, usage, and natural degradation. Carbon dioxide (CO_2_), methane (CH_4_), and syngas can also be used as an efficient substrate for the production of bio-based products, which further aids in the sequestration of greenhouse gases as well as in wastewater treatment (Sciarria et al., 2018; Pérez et al., 2020; Battashi et al., 2021). Additionally, for the cost-effective production of bioplastic, utilization of MMCs (photosynthetic or non-photosynthetic) has been proved to contribute efficiently, owing to the reduced cost of the mixed substrates involved. The usage of MMCs has been reported to facilitate the procurement of continuous culture without any contamination-related peril (Colombo et al., 2017; Figols et al., 2017; de Oliviera et al., 2019).

To accomplish the future agenda of worldwide usage of 100% bioplastic commodities, scientists are targeting the bioplastic production from various microorganisms. MMCs have been widely explored for optimizing their culture conditions, growth, bioplastic accumulation potential, and downstream processing techniques. Despite of great potential of MMCs for bioplastic accumulation, its commercialization is economically not feasible. Thus, it is crucial to thoroughly explore the MMCs for their full potential in terms of PHA production as well as other applications to reduce the cost of scale-up and downstream processing. Tailor-made consortia of microbes can be one such approach in this direction. Therefore, more basic information of the mechanism involved behind the synthesis of precursors of bioplastic in microbes, different critical factors, and unique characteristic of individual microbes in PHA synthesis is required. The present status of bio-based plastic in the market needs to be contemplated to unfold the efficiency of microbes yet not explored for bioplastic production. Hence, this review sheds light on the much-needed route exploration for utilization of microbes, specifically bacteria, algae, and MMCs, in the form of productive cell factories for sustainable bioplastic production on a commercial scale along with the development of retail markets of the same. It gives an insight into the status quo methodologies involved in the processing and production of bio-based building blocks constituting bioplastic. It also focuses on the usage of industrial waste effluents and byproducts as the substrate for the growth of pure (bacteria/algae) or mixed microbial cultures (MMCs) to make the process cost-effective and strengthen the phenomenon of circular bioeconomy.

## Insight Into the Dynamics of Bioplastic Production

### PHA Biosynthesis Pathway

Polyhydroxyalkanoate is a metabolite produced by some microorganisms as a storage product, which also acts as a shield against adverse environmental stress. Enhanced metabolic engineering methodologies have paved the way for exploring and exploiting various PHA biosynthesis pathways, which leads to the formation of different modified PHAs or bioplastics with specific material properties, beneficial for biomedical and industrial applications, such as high-value medical equipment (including sutures and drug delivery), or low-value materialistic bioplastic (cosmetics, coatings, food packaging, etc.; Keskin et al., 2017; Zhang et al., 2018).

Polyhydroxyalkanoates are quite essential building blocks for biodegradable plastic production. PHAs are classified into three categories based on their chain length, including short-chain-length PHAs (C3-C5), medium-chain-length PHAs (C6-C14), and long-chain-length PHAs (C14 and above; Brandl et al., 1988; Steinbüchel and Wiese, 1992; Witholt and Kessler, 1999; McChalicher and Srienc, 2007).

However, out of different types of PHAs produced, PHB (poly-3-hydroxybutyrate), a short-chain-length PHA, has been the most widely characterized and explored bioplastic due to its early discovery in the year 1926 (Lemoigne, 1926). PHB copolymers are synthesized by bacteria when various substrates are introduced interactively and are anticipated to result in the formation of bioplastic composed of either of two types of monomers, i.e., 4-hydroxybutyrate (4HB) and 3-hydroxy valerate (Anderson and Dawes, 1990; Byrom, 1992).

**Figure 1:**
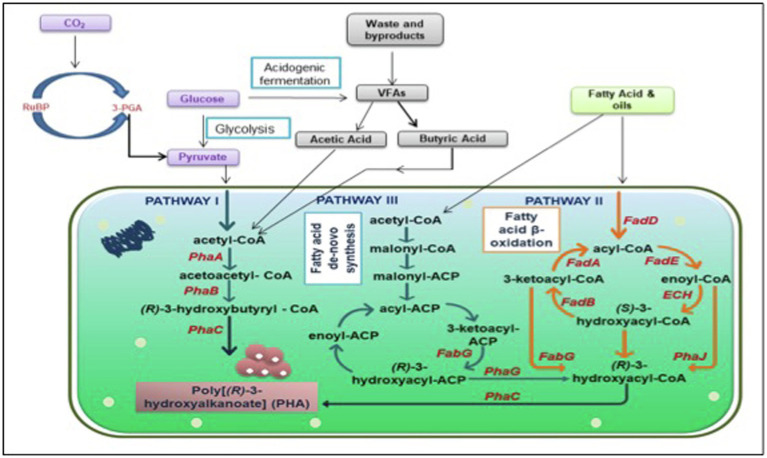
Graphical illustration of interactive PHA biosynthesis pathway from different metabolites. PhaA: β-ketothiolase; PhaB: acetoacetyl-coenzyme A reductase; PhaC: PHA synthase; FabG: 3-ketoacyl acyl carrier protein (ACP) reductase; FadD: fatty acyl-CoA synthetase; FadA: 3-ketoacyl-CoA thiolase; FadE: acyl-CoA dehydrogenase; ECH: enoyl-CoA hydratase; FadB: 3-hydroxyacyl-coa dehydrogenase PhaJ: enoyl-coenzyme A hydratase; PhaG: (R)-3-hydroxydecanoyl-ACP: CoA transacylase; RuBP: Ribulose 1,5-bisphosphate; 3PGA: 3 phosphoglyceric acid; CO_2_: Carbon dioxide; and VFAs: Volatile fatty acids.

A brief illustration of the mechanism justifying the significance of nutrient-depleted/limited conditions is presented in Figure 1. Presence of ample amount of nutrients to the microbes, the Krebs cycle leads to the production of a high quantity of CoA, which in turn obliterates enzyme *PhaA*, 3-ketothiolase to block the accumulation of PHA. This redirects the flux of acetyl-CoA toward the Krebs cycle to accomplish cell growth and energy production. Conversely, when nutrients including phosphorus and nitrogen are not sufficient and an excess of carbon is added in the microbes’ growth environment, a low amount of CoA is produced, which cannot obliterate the PHA biosynthesis enzyme. Consequently, channelizing the acetyl-CoA toward the PHA biosynthesis pathway stimulates PHA aggregation (Jung and Lee, 2000).

Furthermore, a comprehensive PHA biosynthesis pathway, as shown in Figure 1, comprehends the successive action of three prime enzymes (*PhaA*, *PhaB*, *and PhaC*) responsible for culminating three essential reactions. At first, enzyme *PhaA*, i.e., *β*-ketoacyl-CoA thiolase, eventuates the reaction between two acetyl-CoA molecules for acetoacetyl-CoA formation. Subsequently, an enzyme *PhaB*, which is NADPH-dependent acetoacetyl-CoA dehydrogenase, catalyzes the reduction of acetoacetyl-CoA into (R)-3-hydroxybutyryl-CoA monomer. Eventually, enzyme poly-3-hydroxybutyrate polymerase, encoded as *PhaC*, instigates the polymerization of (R)-3-hydroxybutyryl-CoA monomer into poly-3-hydroxyalkanoate (PHA; Madison and Huisman, 1999).

PHA synthesis is also reported to occur when fatty acids and sugar compounds are subjected to β-oxidation or *de novo* fatty acid biosynthesis pathways (Figure 1; Aldor and Keasling, 2003). The oxidation of carbon sources while neglecting the fatty acid β-oxidation pathway leads to the production of acetyl-CoA and transacylase enzyme (*PhaG*), which further catalyzes the biosynthesis of PHA *via* the fatty acid *de novo* biosynthesis pathway. Whereas, when oxidation of carbon sources *via* the fatty acid β-oxidation pathway takes place then enzyme (R)-specific enoyl-CoA hydratase, *PhaJ* catalyzes the oxidization of enoyl-CoA to (R)-3-hydroxy acyl-CoA, which eventually acts as a target precursor for enzyme *PhaC*, PHA synthase, for PHA biosynthesis (Hoffmann et al., 2002; Muhammadi et al., 2015).

There are several other intertwined pathways aside from the three major pathways discussed above. Various anabolic and catabolic reactions take place inside the cells, which contributes in the production of PHA using different precursors. PHB being the most studied scl PHA does not limit the probability of production of numerous other PHAs. Raw material for conventional plastic is cheap to match the cost. Putative pathways have been a foundation of PHA synthesis strategies but to succeed in the battle between conventional plastic and biodegradable plastic, there is need to look at some non-intuitive pathways and focus on those non-competitive substrates. Precursors like amino acids, VFAs, and even greenhouse gases can contribute to PHA production. Bacteria can accumulate up to 80% of their dry cell weight when fed with carbon sources (Budde et al., 2011). Several other bacteria mentioned in Table 1 and section 3 can utilize different carbon sources and accumulate PHA but valorization of these carbon sources are species-specific (García et al., 1999). Few other precursors could be volatile fatty acids, such as acetate, butyrate or propionate, amino acids, and gases like carbon di oxide or methane (Figure 1).

**Table 1:** Different types of PHA accumulated by bacteria under varying substrate supplementation.

Bacterial Strain	Substrates Used	Composition of polymer	Accumulation (% DCW)^#^	References
*Bacillus* sp.	Sucrose, Seawater, Glucose	P(3HB)	20–60	Floccari et al., 1995; Wu et al., 2001; Dhangdhariya et al., 2015
*Aeromonas hydrophila*	Oleic acid, Lauric acid, Gluconate	P(3HB-*co*-3HHx)	15–45	Lee et al., 2000; Qiu et al., 2005
*Ralstonia eutropha/ Cupriavidus nectar*	Coconut Oil, Crude palm kernel oil, Glucose, Fructose, 4-hydroxybutyric acid, Propionic acid, Sodium octanoate, Jatropha oil	P(3HB) P(3HB-*co*-4HB) P(3HB-*co*-3 HV) P(3HB-*co*-mcl-PHA)	20–80	Jung et al., 2000; Green et al., 2002; Wong et al., 2012; Batcha et al., 2014; Obruca et al., 2014
*Alcaligenes latus*	Sucrose	P(3HB) P(3HB-*co*-3HP)	More than 75	Gahlawat and Srivastava, 2017
*Escherichia coli*	Glucose, Xylose	P(3HB)	70–80	Nikel et al., 2006; Nduko et al., 2013
*Pseudomonas putida*	Oleic acid, Glucose, octanoic acid	P(3HB) mcl-PHA	Upto 30	Kitamura and Doi, 1994; Le Meur et al., 2012

# %DCW, % Dry Cell Weight.

PHA, Polyhydroxyalkanoate; P(3HB), poly(3-hydroxybutyrate); P(3HB-co-3HHx), poly(3-hydroxybutyrate-co-3-hydroxyhexanoate); P(3HB-co-4HB), poly (3-hydroxybutyric acid-co-4-hydroxybutyric acid); P(3HB-co-3 HV), poly(3-hydroxybutyrate-co-3-hydroxy valerate); P(3HB-co-3HP), poly(3-hydroxybytyrate-co-3-hydroxypropionate); mcl-PHA, medium-chain-length polydroxyalkanoate.

Significance of metabolic pathways in the production of any biotechnologically important metabolites is known worldwide; however, detailed know-how of every reaction, its reactants, and products along with the knowledge of enzymes involved needs to be gathered primarily before targeting the production of the metabolite of interest. PHA being a type of bioplastic precursor, the understanding of its biosynthesis pathway gives an insight into the nature of catalyst involved, role of varying physico-chemical factors, and even about the type of nutrient media required for diverting the carbon flux toward increased production of PHAs. Another scientific boom in the last decade has been machine-learning approach. With the help of already present database and pathways, various algorithms can be used and analyze the optimum culture conditions related to the selected host (Li et al., 2020). Genome-scale study can also help selecting the organism suitable for specific application (Nobu et al., 2014; Nazem-Bokaee and Senger, 2015).

The diversion of carbon flux in response to diverse environmental stress conditions can be determined by studying the PHA production pathway and its understanding will further aid in optimizing the growth conditions of the microbe or will help in carrying out the genetic manipulation, while targeting upregulation or downregulation of genes involved in the formation of reactants, products, or an enzyme. A deep comprehension of intuitive and non-intuitive metabolic pathway will aid in targeting the raw material in conjucture with the organism and also scrutinizing organisms for a tailor-made consortium for soaring PHA production.

### Factors Influencing Bioplastic Precursor Accumulation

Establishing a sustainable and commercial process for bioplastic production has two main components: quantity and quality of accumulated precursor. High quantity will ensure sustainability of the process but on the other hand, if the properties, which determine equivalence of bioplastic with conventional plastic, will be surpassed, it will fail to make its place in the market.

Properties like crystallinity, glass transition temperature, thermal stability, and melting temperature determine the quality of the polymer (Ten et al., 2015). Homopolymers often suffer from the disadvantage of being brittle; hence, production of copolymers is sought to overcome the hurdle (Bhati and Mallick, 2015). In this section, factors that affect the accumulation of bioplastic precursors are discussed.

Primarily, strain selection plays a very important role in determining the percentage and type of bioplastic precursor being produced. The chemical and physical properties of the bioplastic have been analyzed to be highly dependent on the type of PHA accumulated by different strains. *Alcaligenes latus* has been reported to produce scl PHA, whereas mcl-PHA is commonly produced by *Pseudomonas putida* (Wang and Lee, 1997; Sun et al., 2007).

Another important factor that affects the PHA accumulation potential of microorganism is the composition of nutrient media. The deficiency of essential nutrients or supplementation with waste (rich in carbon sources, amino acids, or fatty acids) has been delineated to influence PHA production. Even the mechanical properties of PHA material get modified based on the nutrients present in the media and it eventually characterizes the use of bio-based material in different fields of biotechnology. PHB yield of *Bacillus megaterium* subjected to various physico-chemical parameters was investigated by Mohanrasu et al. (2020) and concluded that optimization of parameters, such as pH, nitrogen, and carbon sources, needs to be optimized for each bacterial species for the productive accumulation of good quality PHA.

Various nutrients and physico-chemical factors play a role in the biosynthesis pathway of PHA accumulation (Figure 1). Three key enzymes of PHA accumulation are phaA (ketothiolase), phaB (Acetoacetyl reductase), and phaC (PHA synthase). Several copies of PHA synthase gene have been reported over 30 genera of bacteria. All of these genes produce different types of PHA synthase, which determines the conversion of substrate into PHA. It also determines the molecular weight of PHA being produced (Rehm and Steinbüchel, 1999). Molecular weight of PHA determines the crystallinity and thermal stability of bioplastic (Laycock et al., 2014). PHA synthase has three classes that produce PHA with different molecular weight. Class I of PHA synthase produces high molecular weight PHA ranging from 500 kDa to a few millions, class II produces 50 kDa to 500 kDa, whereas class III PHA synthase product’s molecular weight ranges in between (Rehm and Steinbüchel, 1999). High molecular weight also increases young’s modulus which determines the elasticity of the bioplastic (Domínguez-Díaz et al., 2015). Few studies have reported influence of different carbon sources on molecular weight. *Paraburkholderia xenovorans* LB400 was grown in three different carbon sources namely, glucose, xylose, and mannitol. The study concluded that PHB produced with glucose and xylose had similar molecular weight_,_ which is near to commercially available PHB, whereas PHB produced in mannitol supplementation had double M_w_ as compared to control and other carbon sources. The study also concluded that carbon sources not only affect the quantity of PHA production but also the molecular weight of bioplastic (Sanhueza et al., 2020).

Furthermore, the PHB accumulation capacity of carbon-sequestering microalgae, *Botryococcus braunii*, has been evaluated and validated statistically by optimizing different parameters, including temperature, pH, and substrate concentration of sewage wastewater. To some extent, these process parameters were found responsible for diverting the carbon flux toward the enhanced production of PHB. Usage of sewage wastewater as an inexpensive nutrient substrate for algae-based bioplastic production indicated that this strategy could be adopted at an industrial scale for economically feasible and beneficial large-scale PHB production. Microalgae, *B. braunii* was claimed and recommended as a potential candidate for PHB production (around 20% of dry algal weight), which can be used in the pharmaceutical industry. NADH plays a major role in the assimilation of pentose and hexose sugars. Activation of glucose transporters releases NADH, which in turn activates the accumulation of PHA *via* inducing conversion of acetyl coA (Kavitha et al., 2016a,b).

Likewise, *Chlorella fusca* LEB 111 microalgae have also been examined experimentally for improved PHB production by optimizing the requirement of pentose sugar substrate, photoperiod, and light intensity factors, which contribute significantly to the accumulation of PHB. It was demonstrated that 28 μmol photons m^−2^ s^−1^ of light intensity, 6 h exposure to light, and xylose supplementation induced the microalgae to synthesize 17.4% (w/w) PHB. Therefore, deviation in the light periodicity integrated with varying luminous intensities and involvement of pentoses in the metabolic pathway has proven to stimulate and boost the PHB accumulation capacity of *Chlorella*. The uptake of organic carbon is highly dependent on photoperiod and light intensity. Light phase triggers the enzymes responsible for autotrophic growth hence blocking the utilization of carbon sources but during the dark cycle of mixotrophy, it starts assimilating organic carbon hence converting the flux toward PHA synthesis (Cassuriaga et al., 2018).

Irrespective of the significance of either of the microbes, i.e., bacteria or algae in the production of bioplastic, the accumulation of metabolite in the cell along with the growth of microorganism has been delineated to depend highly on various physico-chemical parameters and environmental conditions present at the time of cultivation of microbes. Physical and chemical parameters, such as type of fermentation, type of growth conditions, time of harvesting, aeration, presence or absence of oxygen, pH, temperature, light intensity and the composition of nutrient media, have been studied immensely by researchers for optimizing maximum biomass growth and PHA accumulation of microbe of interest. However, the type of microbe and its strain influences the quality and quantity of the bioplastic produced. Individual microbial cultures will limit the quality and quantity of produced bioplastic; therefore, different algal and bacterial strains need to be studied and the influence of various influential parameters needs to be optimized.

## Prospects of Bioplastic Production in Pure Cultures

### Bacterial Cell Factories

Bacteria are the supreme cell assemblies producing high molecular weight biomolecules, assembled by the linkage of building blocks, such as hydroxy fatty acids, sugars, and amino acids *via* catalytic enzymes, to formulate a diverse range of bioplastic with varying mechanical and chemical attributes. Biomolecules, such as polysaccharides (consist of sugar moieties intertwined by glycosidic bonds), polyamides (comprise of amino acids intertwined by peptide linkages), polyesters (consist of ester-bond linked hydroxy fatty acids), and polyphosphates (composed of inorganic phosphates connected by anhydride bonds), are produced by different bacterial species. These have been made and utilized substantially on a commercial scale while considering the recent advances in bioengineering methods and molecular techniques (Muhammadi et al., 2015; Schmid et al., 2015; Wang et al., 2016; Wendisch et al., 2018). French researcher Maurice Lemoigne discovered the first-ever known bioplastic – PHB in 1926 while prospecting the bacterium *Bacillus megaterium* (Lemoigne, 1926). PHAs are linear polyesters produced and deposited as water-insoluble, amorphous, and intracellular spherical inclusions or granules of 0.2–0.7 mm *via* PHA metabolism (Girotto et al., 2015). Wide range of bacteria (Gram-negative and Gram-positive) is reported to synthesize PHA, with varying composition, resulting in immensely diversified materialistic properties (Schubert et al., 1988; Steinbüchel et al., 1992; Rehm et al., 1998; Rehm and Steinbüchel, 1999; Rehm, 2003).

PHAs are highly popularized on a commercial scale because they resemble oil-based plastic properties, such as their thermoplasticity, depicted by 30 to 70% crystallinity and 50–180°C melting temperature, making them a perfect fit in the bioplastic industry as biodegradable and renewable bioplastic. A detailed description of several bacteria producing different types of PHAs under the supplementation of varying substrates has been listed in Table 1 (Jendrossek, 2009; Kai and Loh, 2014; Favaro et al., 2019).

According to the previous research done on PHA, it is postulated that as compared to the PHA accumulation in plants, which is less than 10% dry cell weight, bacteria have the potential to accumulate the magnificent amount of PHA (90% dry cell weight; Steinbüchel and Lütke-Eversloh, 2003; Verlinden et al., 2007). It has been elucidated that based on the requirement of varying constraint conditions, PHA-producing bacteria can be categorized into two groups: non-growth assisted and growth assisted. The bacterial strains, such as *Protomonas extorquens*, *A. eutrophus*, *Cupriavidus necator*, *Protomonas oleovorans*, *Pseudomonas* sp. *2F*, and *Methylmonas extorquens*, are some of the microbes that aggregate enhanced PHA in nutrient-deprived/limited (including, nitrogen, phosphorus, magnesium, and sulfur) and excess carbon supplemented environment (Braunegg et al., 1998; Ciesielski et al., 2010), whereas another class of bacteria, such as *Alcaligenes latus*, *Paracoccus denitrificans*, *Azohydromonas lata* DSM 1122, and *Pseudomonas putida* GPol ATTC 29347, synthesizes PHA without the need of nutrient-limited conditions (Byrom, 1994; Hartmann et al., 2006; Koller et al., 2010; Anjum et al., 2016).

The pure culture of bacterial strains, such as *Bacillus*, *Azotobacter*, *Nocardia*, *Alcaligenes*, *Pseudomonas*, *Ralstonia eutropha*, and *Rhizobium*, is considered extensively for commercial production of PHB (Chaudhry et al., 2011; Jiang et al., 2011a; Chandani et al., 2014). Budde et al. have reported that bacterium *Cupriavidus necator* H16 (earlier known as *Ralstonia eutropha*), when supplemented with fructose as carbon supply, can potentially accumulate 79% of the dry cell mass of PHB under optimal conditions (Budde et al., 2011).

Concurrently, the commencement of genetic engineering approach for refining all the stages of the PHA production process, mostly related to the types of substrates up taken and utilized, has laid a cornerstone in the field of bioplastic production. For instance, intra-genus PHA genes cloned and expressed in *E. coli* from *Azotobacter* sp. have shown up to 72% PHB accumulation with corn steep liquor as carbon and nitrogen source. There are different examples of genetic manipulation done to achieve an elevated amount of good quality PHA without compromising the cell growth (Povolo et al., 2010; Liu et al., 2011; Chen and Jiang, 2018; Ouyang et al., 2018).

In spite of extensive research and genetic manipulation from more than three decades, the cost of bioplastic has yet not been able to match that of conventional plastic, the key rationale being high cost of the substrate. For a cost-effective production of PHA, researchers have reckoned photosynthetic bacteria. Photosynthetic bacteria can be divided into cyanobacteria, purple bacteria, and green bacteria. Purple bacteria are anoxygenic bacteria, which means they do not produce oxygen during photosynthesis. They use sulfur (purple sulfur bacteria PSB) or hydrogen or ferrous ions (purple non-sulfur bacteria PNSB) as an electron acceptor during photosynthesis (Mothersole et al., 2018). They can be used for protein accumulation, heavy metal absorption as well as PHA production (Talaiekhozani and Rezania, 2017; Alloul et al., 2019; Foong et al., 2019).

Both PSB and PNSB can accumulate PHA under autotrophic and heterotrophic conditions. In a study, 12 strains containing nine PNSB and three PSB were exposed to organic and inorganic carbon as well as a mixture of both under nitrogen starvation and PHA accumulation was observed. Organic carbon yielded a higher accumulation of PHA as compared to inorganic carbon. *Rhodovulum visakhapatnamense* accumulated up to 30% accumulation and *Rhodovulum sulfidopilum* when exposed to only acetate in seawater accumulated 9% PHA (Higuchi-Takeuchi et al., 2016). PNSB prefer VFAs as a precursor for the production of PHA (Higuchi-Takeuchi and Numata, 2019). Acetate is considered the best precursor for PHB synthesis, having least steps involved in the conversion. Other VFAs like propionate, butyrate, and valerate also have a high assimilation rate during the metabolism of PNSB. A proteomic study revealed that VFAs are responsible for rapid and high accumulation of PHA in PNSB; however, bicarbonate is also important in the assimilation of valerate and other volatile fatty acids except for acetate in *Rhodospirillum rubrum* (Bayon-Vicente et al., 2020).

As it has been established before that purple bacteria favor VFAs over other carbon sources, studies have been conducted to find cheaper alternatives of VFAs from waste. Palm olive mill waste has high VFA and chemical oxygen demand (COD) which was utilized by *Rhodopseudomonas* sp. S16-FVPT5 to accumulate 315 mg/l PHB and 2,236 ml/l hydrogen (Carlozzi et al., 2019). They can also valorize high lignin-containing waste after thermal treatment and convert it into 21% PHB of their dry cell weight (Allegue et al., 2021).

It is evident that the bioplastic precursors derived from bacteria prove to be an imperative constituent of bioplastic commodities. There are several plastic industries worldwide, manufacturing bioplastic products from the precursors derived from bacteria, and the products have the potential of replacing fossil-based plastic. However, large-scale production of bacterial bioplastic has started from past few years and to make the process feasible as that of conventional plastic production, more research needs to be performed in this domain. The factors affecting the accumulation of bioplastic precursors, their extraction and purification process, and at last their transformation into bioplastic commodities need to be investigated on a large scale. The evaluation needs to be done to explore the bioplastic production capability of pure strains as well as MMCs. The biorefinery approach for the production of bacterial bioplastic along with other essential bioproducts derived from bacteria needs to be scrutinized and exercised in order to make the process techno-economically feasible. Additionally, systems analysis incorporating LCA and techno-economic analysis must be investigated for determining the sustainability of bacterial bioplastic production.

### Algal Bioplastic

For years, photosynthetic organisms, such as microalgae and cyanobacteria, have been highly explored for their involvement in trapping solar energy and balancing the atmospheric carbon (Ghosh and Kiran, 2017). The crucial life elements, including solar energy, CO_2_, and water, are processed through microalgae metabolism to form bioenergy products. Because of their high cell proliferation rate, microalgae’s conversion efficiency is postulated to be 10 to 50 times higher than that of terrestrial plants. Thus, the microalgal land footprint required for bioenergy production is significantly less than plants. The most prevalent and topical agenda in the field of algal biotechnology is accomplishing the tremendous opportunities catered by algal biomass and their widespread biochemical portfolio. Due to their exceptional potential of assimilating photosynthetic carbon, microalgae are enunciated as a highly efficient form of cell biomass produced and has immense potential to engage in a clean energy future (Kiran et al., 2014). Economic constraints obviate many emerging commercial endeavors, and a lot of such barriers can be conquered by incorporating a diligent conceptual configuration for augmenting the algal biomass values (Laurens et al., 2017).

As a spin-off from algal biofuels, algae are recognized as proficient producers of various exclusive metabolites, such as polyunsaturated fatty acid, carotenoids, phycobilins, polyhydroxybutyrate, and so on, which are of commercial importance in pharmaceutical, nutritional, plastic, cosmetic, and other biotechnology-based industries (Laurens et al., 2017; Martosa et al., 2019; Arun et al., 2020). Additionally, microalgae being a phototroph, consumes inorganic nutrients, such as nitrogen, phosphorus, sulfur, and carbon dioxide along with solar energy for biomass proliferation and cell growth. The growth prerequisites for microalgae are expedient compared to that of heterotrophs, such as bacteria and yeast. Due to the requirement of organic nutrients by bacteria for accumulating imperative metabolites, makes the cultivation model of heterotrophs expensive. It is deduced that while taking into account the production cost of bacterial bioplastic, the largest contributing factor in the hiked production cost is the usage of tremendous amount of organic substrate for fermentation process, followed by the cost and energy-intensive PHA extraction procedures, eventually making the bacterial bioplastic production economically infeasible (Perez-Garcia et al., 2011; Markou and Nerantzis, 2013; Karan et al., 2019). Comparatively, the commercialization of microalgal bioplastic is hindered by the high cost involved in the traditional phototrophic cultivation techniques. Hence, presently, microalgae-derived bioplastic appears to be infeasible compared to the conventional processes but synergized cultivation of microalgae together with the treatment of wastewater paves the way for the reduced commercialization cost. The production of microalgae-emanated bioplastics has been consummated through different research methodologies, including (1) amalgamation of microalgae biomass with bio-based or fossil-based plastics or their additives (2) complete usage of microalgae biomass in the form of bioplastic (3) improving the metabolite (of interest) accumulation capacity of specific microalgal species along with its cost-effective extraction (4) processing microalgae bioplastic while obeying the biorefinery model, and lastly (5) administrating genetic engineering methodologies for the creation of proficient microalgal strain to synthesize an ideal bio-based plastic (Jungmeier, 2014).

#### Accumulation of Bioplastic Precursor in Algae

Bioplastic production from microalgae is considered to be an efficacious strategy for direct carbon entrapment (by mitigating CO_2_ produced from flue gas), accompanied by the acquisition of imperative biochemical constituents, including lipids (Ho et al., 2014; Nakanishi et al., 2014; Anand et al., 2019), proteins (Schwenzfeier et al., 2011), carbohydrates (Ho et al., 2013; Cea-Barcia et al., 2014), and PHA (Roja et al., 2019) that can act as potential bioplastic feedstock. Ultimately, establishing the fact that CO_2_ sequestered by bioplastic-producing microalgae is directly getting entrapped in the form of polymer and not exiting into the environment (Abdul-Latif et al., 2020; Crocker et al., 2020).

In addition to that, supplementation of organic nutrients to activate the organism’s heterotrophic metabolism, along with the addition of varying combinations of crucial nutrients, to accomplish the synthesis of aggravated PHA can effectuate a cost-effective cultivation model for microalgal bioplastic synthesis (Table 2; Dietrich et al., 2017; Silva et al., 2017; Di Caprio et al., 2019a,b; Karan et al., 2019; Abiusi et al., 2020). Conclusively, varying physical and chemical nutrient conditions of microalgae have been recognized to influence the quality and quantity of metabolites being produced stupendously; thus, perturbing conditions can be exploited and scrutinized further for boosting the production of a metabolite of our interest, such as polyhydroxyalkanoate (PHA; Gualtieri, 2001; De Morais and Costa, 2007; Hossain et al., 2008; Noreen et al., 2016). A perspective that starch-based bioplastics could be made from the starch-rich (49% w/w) biomass of microalgae, such as *Chlamydomonas reinhardtii* 11-32A by exercising efficient plasticization through the twin-screw extrusion process was established (Mathiot et al., 2019).

**Table 2:** Algal species accumulating different forms of bioplastic.

Algal Strain	Type of Polymer	Accumulation (%DCW)^#^	References
*Nostoc muscorum*	PHB	31–69	Sharma and Mallick, 2005; Samantaray and Mallick, 2014
*Phaeodactylum tricornutum*	PHB	10.6	Hempel et al., 2011
*Spirulina platensis*	PHB	6.20	Maheswari and Ahilandeswari, 2011
*Botryococcus braunii*	PHB	20–60	Kavitha et al., 2016a, 2016b
*Synechocystis* sp. *PCC 6714*	PHB	37	Kamravamanesh et al., 2017, 2018
*Chlorella fusca* LEB11	PHB	17.4	Cassuriaga et al., 2018
*Microcystis aeruginosa*	PHB	4.38	Abdo and Ali, 2019
*Scenedesmus* sp.	PHB	1–30	García et al., 2021

# %DCW% Dry Cell Weight.

PHB = Polyhydroxybutyrate.

Kato (2019) also reported that triacylglycerol-rich algal biomass of *Chlamydomonas reinhardtii* could be molded, in the crude form, directly into 7 mm bioplastic beads that can withstand compressive stress of up to 1.7 megapascals, thus excluding the cost of extraction and purification processes involved in the bioplastic production. Amidst all the metabolites produced, polyhydroxyalkanoate (PHA) is the highly explored and most expedient building block of microalgae bioplastic due to its easy biodegradation, catalyzed by enzymes (Shi et al., 2012; Noreen et al., 2016; Rahman and Miller, 2017; Karan et al., 2019; Beckstrom et al., 2020). The production of polyhydroxybutyrate from microalgae in high-rate algal pond (HRAP) was analyzed along with their plasticization capacity, and it was concluded that out of two microalgal strains, *Microcystis aeruginosa*, exhibited the highest PHB concentration. However, high-rate algal pond biomass dominated with *M. aeruginosa* showed the highest PHB concentration and was utilized for the production of bioplastic (Abdo and Ali, 2019).

Microalgae being oxygenic photoautotroph can also grow in other modes like heterotrophy or mixotrophy (Gong and Huang, 2020). Cyanobacteria can accumulate PHA in photoautotrophic modes as reported previously under controlled conditions (4.5%) or under phosphate limitation (11%; Panda and Mallick, 2007). Many other reports have also established a relationship with mixotrophic and heterotrophic production of bioplastic by several cyanobacteria like *Nostoc muscorum*, *Synechocystis* sp., and *Microcystis* sp. (Table 2; Bhati and Mallick, 2012; Gopi, 2014; Abdo and Ali, 2019).

Microalgae are now being approached by researchers very rapidly for PHA production as well (Roja et al., 2019; García et al., 2021). But using pure substrate and maintaining pure culture at a large scale for PHA production will still fall short at economic point of view. Hence, inducing non-intuitive pathways *via* physico-chemical stress or adapting different approaches and application of microalgae in PHA production is far-reaching (Chalima et al., 2017; Llamas et al., 2020).

#### Algal Biocomposites

Researchers also addressed the formation of an innovative algal composite comprising of *Chlorella* sp. (hydrophilic, granular, and biofix CO_2_ efficiently) and materials, such as polyvinyl chloride (hydrophobic), polyethylene, and polypropylene. The algal composite is characterized as a composite of high tensile strength and thermal stability due to the presence of *Chlorella* grains and their binding capability (Zhang et al., 1999, 2000a,b; Otsuki et al., 2004). Lee et al. used red algae, *Gelidium elegance*, to produce poly butylene succinate-based biocomposites by the compression molding process. The study suggests that bleached red algae fiber can be incorporated for manufacturing environmental-friendly biocomposites (Lee et al., 2008). Chiellini et al. produced eco-compatible hybrid biocomposites using green algae fibers and polyvinyl alcohol polymers (Chiellini et al., 2008). Intuitively, encapsulation of algal biomass of *Nannochloropsis* sp. and *Spirulina* sp. in thermoplastic polymers, such as polypropylene, polyurethane, and polyethylene, suggested that manufacturing plastic based on algae is a prudent and imperative mechanism to sequester and retain atmospheric CO_2_ fixed proficiently by algae (Shi et al., 2012). Moreover, while considering the biorefinery model of microalgal species, such as *Botryococcus braunii* and *Nannochloropsis gaditana*, integration of residual microalgal biomass left after the extraction of biodiesel with biodegradable polymers poly butylene succinate and poly butylene adipate-co-terephthalate, respectively, revealed that the residual biomass could be scrutinized further to fabricate biocomposites in a cost-effective manner (Toro et al., 2013; Torres et al., 2015).

Furthermore, in pursuance of microalgal bioplastic production, Zeller and colleagues in 2013 illustrated the significance of polymerization of protein-rich microalgae biomass thermo-mechanically to synthesize algae-based bioplastic as well as thermoplastic blends or biocomposites. The biomass of *Chlorella* sp. and *Spirulina* sp. consisting of a high amount of protein, i.e., 58 and 57%, respectively, was integrated with varying glycerol concentrations and mechanical testing of bioplastic was performed through thermo-mechanical molding by preparing ASTM standard “dog bones.” The bioplastic mix produced was further blended with polyethylene polymer and was observed that *Spirulina* was more compatible with polyethylene as compared to *Chlorella*, exhibiting significant mechanical properties, making the blend suitable for commercial usage. However, pure *Chlorella*-based bioplastic, without any blend, displayed better bioplastic properties, such as high tensile strength along with superior extension and loading properties, than pure *Spirulina*-based bioplastic (Zeller et al., 2013). Sabathini et al. (2018) also illustrated that the eco-sustainable plastic film made from pre-treated *Chlorella* and polyvinyl alcohol mixture could perform favorably for manufacturing packaging plastic material due to its increased bioplastic tensile strength as well as elongation percentage.

#### Genetic Manipulation for Microalgal PHB Accumulation

Microalgae *Phaeodactylum tricornutum* was investigated by Hempel et al. for its ability to act as a photosynthetically active bioreactor to synthesize bio-based plastic. For the first time, the solar-powered microalga was proclaimed to serve as an efficient biosynthetic expression factory when the PHB production pathway of bacterium *Ralstonia eutropha* H16 was incorporated into the biochemical pathways or metabolism of *P. tricornutum* to synthesize building blocks of bioplastics, such as PHB. The granular PHB accumulated after bacterial enzyme expression was determined to be 10.6% of the dry algal weight (Peplinski et al., 2010; Hempel et al., 2011).

PHB biosynthesis genes for enzymes PhbB and PhbC derived from bacterium *R. eutropha* were incorporated into microalga *C. reinhardtii* for PHB production. It already had the gene for the enzyme PhbA, essential for PHB biosynthesis. The research illustrated that the algae *C. reinhardtii* was able to accumulate 6 mg/g dry cell weight of PHB after the expression of bacterial genes (Chaogang et al., 2010). The studies mentioned earlier manifest likelihood of formation of a genetically modified microalgae for the production of PHA, but scaling up of a process with GMOs have their own drawbacks circling back to the cost-effectiveness of bioplastic production and commercialization (Beacham et al., 2017). Entailing another approach for uncovering the true potential of microbes for PHA synthesis is a must.

#### Algal Bioplastics Predicaments and the Upcoming Resolutions

The synthesis of algae-based bioplastic comes with many challenges, and unraveling the complications needs to be the prime target for designing the upcoming research initiatives. For instance, bioplastic materials comprising algae-derived lipids are proclaimed to emit displeasing odors, thus confining the range of products of such bioplastics. Similarly, the characteristic of sugars or carbohydrates to form an aggregate inside the algal biomass leads to prolonged production and extraction steps, making it time and money-consuming. However, regardless of these hindrances, mechanical characteristics exhibited by algae-derived bioplastics are much better than their fossil-based analogs. The superior quality is attributed to the process of microalgae protein getting stretched and intermingled into the polymer matrix, establishing the significance of protein-rich microalgal biomass to produce high-quality bioplastic. Therefore, the biorefinery model for microalgae cultivation can be incorporated proficiently to synthesize lipid or carbohydrate-based biofuels and simultaneously accumulate integral monomer units formulating into bioplastics, thus reducing the extraction and manufacturing cost involved during algae-based plastic production (Beckstrom et al., 2020).

In a nutshell, algal bioplastic production is still in its infancy as compared to bacterial bioplastic production. Besides, the waste to bioenergy conversion potential of algae has not been explored to the fullest for the treatment of waste (wastewater, food wastewater, and industrial waste) coupled with the production of bioplastic precursors. Even co-production and extraction of different bioproducts in an algal biorefinery have been researched evidently, but the accumulation of bioplastic in a biorefinery setup has not yet been reviewed. The potential of different algal strains, their growth, utilization of waste (exhaust CO_2_ as well wastewater), and co-production of bioplastic along with other valuable products in a biorefinery setup needs to be envisioned to accomplish the objective of 100% bio-based plastic.

Consequently, the entrapment and storage of carbon dioxide permanently in algal biomass have also been achieved by encapsulating other polymers (biodegradable or non-biodegradable) entities into the algal blends of thermoplastic nature. Equivalently, employing wastewater as a nutrient substrate for algae-based plastic production has also resulted in a beneficial strategy for synthesizing biodegradable bioplastics in an economically efficient manner. Indeed, carrying out concurrent research while considering algae as a promising and harmless bioplastic candidate can aid in diminishing the dependency on fossil fuels, improving the quality of plastic produced, and can help in minimizing the negative impact of petroleum plastics on the environment.

### Scale-Up and Downstream Processing of Microbial Bioplastic in Pure Cultures

Although microbial community has been determined as a proficient candidature for bioplastic production; however, just optimizing the growth and accumulation of bioplastic precursors at lab. Scale is not sufficient enough for commercialization of bioplastic material. Scaling up of lab-scale parameters on pilot scale have frequently let down, reason being the added factors related to type of pilot-scale plant. Pilot-scale plant includes two categories: closed bioreactors and open pond systems.

Closed bioreactors are often utilized for upscaling axenic cultures. It is easy to maintain purity and controlled conditions in closed reactors, but additional costs to operate the reactor have a negative impact on the cost of final product (Alam et al., 2020). Another factor adding to the cost is substrate. Using pure substrate at pilot scale is not feasible from an economic point of view. To overcome these factors, many studies have performed and introduced various cheap substrates for PHA production (Bhagat et al., 2020; Riedel and Brigham, 2020). Operational conditions like agitation, and aeration impacts growth as well as PHA accumulation inside the reactor. *Halomonas campisalis* MCMB-1027 was grown in a 14 L reactor and accumulated 41% PHA under optimum agitation and aeration. Optimized dissolved oxygen level (DO) further increased accumulation up to 56%. This optimization helped developing a correlation for upscaling up to 120 L fermentor (Kshirsagar et al., 2013). Optimizing all the operational conditions can be time consuming, model-based approaches can resolve limitations (Papapostolou et al., 2019). Closed photobioreactors are often used for photosynthetic microorganisms. Flue gases can be used as a substrate to trigger PHA accumulation (Troschl et al., 2017). A semi-continuous tubular photobioreactor was used to grow *Synechocystis* sp. CCALA192, CO_2_ was used as substrate, and 12.5% PHB was accumulated in the semi-continuous mode (Troschl et al., 2018). Byproducts like glycerol, grape pomace, and molasses can also serve the purpose (Nighat Naheed, 2012; Follonier et al., 2015; Volova et al., 2019).

High-rate algal ponds often used to grow microalgae, utilizes waste water as substrate (Chakravarty et al., 2010; Simon et al., 2016; Abdo and Ali, 2019). It has a low operational cost as compared to closed reactors but maintaining axenic cultures is tough task, especially when wastewater has high COD and VFA content. Scaling up mixed culture is far better due to low maintenance and wide range availability of substrate choices and in tailor-made consortium, microorganisms based on their attributes and compatibility can be selected for soaring PHA production.

Even pilot-scale production of PHB accumulated within the cyanobacteria has been known to be difficult due to the contamination-related challenges which eventually hinder the purity of the type of bioplastic produced (Troschl et al., 2018). It has been postulated that a distinct strategy needs to be devised for large-scale cultivation of bioplastic. Thus, while optimizing upstream processing steps at lab-scale, pilot-scale approach should also be looked into extensively, followed by the investigation of appropriate extraction strategy. A sustainable bioplastic purification process that is cost-effective has a lower impact on the environment and higher conversion efficiency as compared to the traditional methodology. As the cost of different steps of bioplastic downstream processing elevates the overall bioplastic manufacturing cost; therefore, detailed scrutinization of different types of extraction and purification techniques taking part in the process should be taken into account for facilitating commercialization of bio-based plastics.

Downstream processing has also been known to contribute substantially in determining the economic status of bioplastic production owing to high accumulation of PHA inside the cell and lower yield of product extracted; therefore, different extraction and purification strategies also need to be investigated to make the process economically feasible and environmentally sustainable (Lorini et al., 2021).

While extracting and purifying bioplastic precursors from the microbe, several factors need to be considered for discerning the strategy to be adopted for economical downstream processing. Parameters, such as type of PHA-producing microbe, yield, and type of bioplastic, and the purity of the product to be extracted help in deciding the extraction procedure. Eventually, the extraction and purification methodology employed not only influences the type of bioplastic material produced but also delineates the environmental and economical consideration of the same (Koller et al., 2013).

A comparative analysis of various strategies available for PHA extraction has been done by Kurian and Das, 2021. In the following study, they have mentioned chemical and biological extraction processes and concluded biological process is better than the chemical approach. In another study with a similar aim suggested choosing extraction process on the basis of end product (Kunasundari and Sudesh, 2011).

Additionally, LCA and techno-economic analysis of various PHA recovery processes have been performed and illustrated that the usage of solvent leads to a hike in the production cost of bioplastic and should be persuaded only while employing solvents obtained from biorefinery setup or should be performed when absolute form of bioplastic precursor is required. Likewise, economical and environmental impact of every methodology involved in the downstream processing of bioplastic precursors needs to be exercised for attaining sustainable bioplastic production design (del Oso et al., 2021).

## The Quintessence of Mixed Microbial Culture in Bioplastic Production

The utilization of pure microbial cultures for improved PHA production requires the supplementation of costly co-factors, vitamins, and organic substrates to strengthen the microbial metabolic processes, accompanied by the strict process control framework initiated by sterilization of equipment and growth media. All these parameters and requirements add-on to bioplastic production cost, thus obliterating the commercialization of bio-based plastic. In order to overcome these economical and technical hurdles, the administration of MMCs or photosynthetic microbial cultures (PMC) for the production of building blocks of bioplastic, such as Polyhydroxybutyrate or polyhydroxyvalerate, has been proposed. The MMC utilization caters to the reduced bioplastic production cost owing to the consumption of inexpensive heterogeneous feedstocks complemented with the open cultivation conditions, neglecting the need for sterilization or stringent control regulations. Volatile fatty acids including, butyrate, propionate, acetate, and valerate obtained from the fermentation of sewage streams are known to be the best precursors utilized by MMCs for PHA production (Albuquerque et al., 2011). Certainly, the financial supremacy and significance of MMCs over pure cultures for PHA production have also been illustrated by the comparative financial analysis and LCA of MMC’s PHA production (Gurieff and Lant, 2007). Nevertheless, apart from the economic outlook, wastewater or sewage stream usage as an alternative to the nutrient substrate by MMC’s along with the production of biodegradable plastic has contributed to the diminished environmental footprint of bioplastic production.

However, to generate an elevated amount of PHA, the microbial genera present in the MMC needs to be specified and optimized. For the selection of microbes, the culture has been transiently fed by obeying the feast and famine regime, which comprises of an intermittent feeding strategy, delineated as the enrichment of the culture with external carbon substrate (Feast) for a particular period of time, followed by the depletion of the carbon substrate (Famine) in the alternative time period. These feast and famine cycles are repeatedly practiced, which favors cell growth, as well as accumulation of storage molecules, such as PHA during the feast cycle and later gets consumed during the famine cycle. Thus, microorganisms having high PHA storage capacity will ultimately survive and grow, becoming the microorganism of choice for inclusion in the MMCs (Dionisi et al., 2006; Serafim et al., 2006; Albuquerque et al., 2007, 2010a,b; Oliveira et al., 2017).

Furthermore, the operating conditions and the composition of the substrate also play a significant role in the selection of diverse microbial species (Jiang et al., 2011a,b,c). Different researchers have worked on the varied substrate preferences of these organisms as presented in Table 3, which provide significant knowledge that might be used further to optimize PHA accumulation by tweaking the microbial community’s assembly (Jiang et al., 2011a; Albuquerque et al., 2013; Marang et al., 2013; Pardelha et al., 2013; Carvalho et al., 2014). Subsequently, the system’s operating parameters, including pH, temperature, feeding strategy, sludge retention time, a feast to famine ratio, and organic loading rate, have also been observed to significantly affect the PHA composition and content by influencing the culture conditions of MMC (Dionisi et al., 2006; Johnson et al., 2009, 2010; Villano et al., 2010; Albuquerque et al., 2011; Jiang et al., 2011c; Chen et al., 2013; Wang et al., 2013; Carvalho et al., 2014).

**Table 3:** PHA accumulation by MMCs under the supplementation of varying substrates.

Substrate	Bioplastic monomer	PHA Accumulation (%DCW)^#^	References
Crude Glycerol	PHB	49–60% dcw	Dobroth et al., 2011
Fermented Dairy Manure	P(3-HB-co-3-HV)	22.5–90.7% dcw	Coats et al., 2016
Fermented cheese whey with acetate pulse	P(3-HB-co-3-HV)	30%/VSS	Fradinho et al., 2019
Acidified hardwood spent sulfite liquor	P(3-HB-co-3-HV)	44.5% dcw	Pereira et al., 2020
Volatile Fatty acid	P(3-HB-co-3-HV)	44% dcw	Guerra-Blanco et al., 2018
Sucrose	P(3-HB-co-3-HV)	23.8 mg/L/Day	Löwe et al., 2017
Fermented Domestic Wastewater	P(3-HB-co-3-HV)	30.8%/VSS	Almeida et al., 2021

# %DCW = % Dry Cell Weight.

PHB = Polyhydroxybutyrate, VSS = Volatile suspended solids; and P(3HB-co-3 HV) = poly(3-hydroxybutyrate-co-3-hydroxy valerate).

As per the aforementioned literature, besides bacteria, algae have also been in the league of PHA production. Based on aforementioned facts, the algae-bacteria consortiums have been ascertained and reported to have the potential for wastewater treatment accompanied by the production of different biotechnologically essential bioenergy products (Ogbonna et al., 2000; Safonova et al., 2004; Van Iersel, 2009; Perez-Garcia et al., 2011).

According to the literature study done, the highest energy-intensive prerequisite of MMCs recognized is the supply of proper aeration for fulfilling the requirement of oxygen (an electron acceptor) because the PHA content accumulated in an aerobic environment (75%) is substantially high as compared to the PHA content gathered in anaerobic/aerobic environment (37%). However, the requirement of aeration ultimately increases the operation cost involved in the PHA production by MMCs (Dionisi et al., 2004; Dai et al., 2007; Rosso et al., 2008; Bengtsson, 2009; Albuquerque et al., 2010b).

While looking into the PHA production efficiency of pure bacterial species and mixed microbial consortia, an example of *Ralstonia eutropha* can be considered. *R. eutropha* independently under the supplementation of VFAs derived from food waste is known to accumulate approximately 52% PHA copolymer (Bhatia et al., 2019). Although *R. eutropha* cannot assimilate sucrose in the form of carbon sources, however when synthetic mixed microbial consortia composed of *Bacillus subtilis* and *R. eutropha* is grown in the presence of sugarcane sugar then it leads to the accumulation of about 66% PHA copolymer. Therefore, it suggests the importance of MMCs over the pure bacterial culture for the purpose of PHA production (Bhatia et al., 2018). The biosynthesis of PHB by MMC supplemented with the crude glycerol (obtained as a byproduct from the production of biodiesel) was evaluated by a group of scientists and illustrated that due to the deficiency of macronutrients in the presence of crude glycerol, accumulation of PHB is elated. They projected that on scaling up, about 20.9 tons of PHB could be produced every year by MMCs while processing 10 million gallons of biodiesel every year (Dobroth et al., 2011).

Therefore, to vanquish this obligation, Fradinho et al. (2013a) culminated the enrichment of photosynthetic mixed culture comprising of bacteria and algae consortia by observing the accumulation of PHA by bacteria in the feast cycle followed by its consumption in the famine cycle while using the oxygen generated by the photosynthetic algae, thus excluding the need of aeration provided externally. The PHA accumulated by algae-bacteria consortia was reported to be 20% PHA per volatile suspended solid (VSS) when the culture was supplemented with acetate in the form of a carbon source.

It has been proven by the previous studies that oxidation of reducing molecules like NADP and NADPH is required for conversion of carbon source into PHA. In the absence of oxygen, these reducing molecules will never be oxidized, and carbon source conversion to PHA will not be initiated. Hence, justifying the quintessence of photosynthetically mixed culture (composed of algae and bacteria consortia) for ameliorated polyhydroxyalkanoate production (PHA; Fradinho et al., 2013a,b, 2014, 2016).

**Figure 2:**
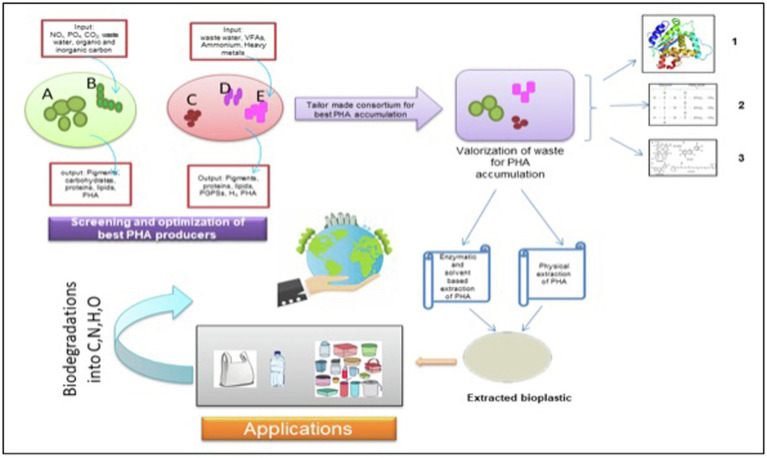
An illustrative biorefinery approach for tailor-made consortium of photosynthetic microorganisms. Microalgae; B- Filamentous Cyanobacteria; C- Photosynthetic bacteria 1; D- Photosynthetic bacteria; and E- Photosynthetic bacteria. 1. Protein; 2. Triacylglycerides, and 3. Pigments. NO_x_: Nitrates, PO_4_ – Phosphate, CO_2_: Carbon dioxide; VFAs: Volatile fatty acids; PGPSs: Plant growth promoting substances; H_2_: Hydrogen; PHA: Polyhydroxyalkanoates; C:Carbon; N: Nitrogen; H: Hydrogen; and O: Oxygen.

Weiss et al. too designed a synthetic consortium comprising of a photoautotroph and a chemoautotroph. The synthetic consortia were light-driven, and the heterotrophic *Halomonas boliviensis* bacterium was capable of metabolizing sucrose secreted by algae, *Synechococcus elongatus* CscB. It was reported that the bacteria in consortia were able to accumulate about 31% dry cell weight of PHB, with the productivity of 28.3 mg PHB L^−1^D^−1^ (Kourmentza et al., 2017; Weiss et al., 2017). In a nutshell, an upcoming paradigm of embracing the importance of MMCs for the production of bioplastic, especially PHA, should be practiced to ensure sustainable and economically feasible bioplastic production on a commercial scale (Figure 2).

On summarizing the positive impact of photoautotrophic cultivation of algae, i.e., no specific requirement of agricultural land, high CO_2_ sequestration ability, photosynthetic efficiency, and treatment of waste material prove to be economically proficient over the heterotrophic growth of bacteria. However, even the maintaining axenic cultures of algae on a large scale has been known to be economically infeasible due to maintenance cost of the optimal physico-chemical parameters for the production of PHA. Therefore, to overcome all the challenges discussed previously, MMCs composed of either different chemoautotrophic bacteria or that of algae and phototrophic bacteria need to be practiced and explored further for making the PHA production process economically feasible. Not only different microbes participating in the mixed consortia need to be investigated for their PHA yield but varying physical and chemical parameters affecting the accumulation capability of the consortia also need to be optimized for obtaining supreme yield keeping in view the large-scale production of bioplastic.

## Existing Commercial Status Quo of Bioplastics

The global upsurge and advancement in the bioplastic market are attributed to its sustainable production protocol (compared to conventional plastic) and its widespread applications in different industrial fields, such as food services and packaging. Moreover, increasing awareness regarding the benefits of biocompatible and biodegradable plastic, formulation of government norms to stimulate and acknowledge eco-friendly techniques, and ever-augmenting fossil-fuel prices have invigorated the commercial diversion toward the production of bioplastic elements.

The worldwide spread of bioplastic business is still subordinate as compared to the petroleum-based plastic market. Different bioplastic-producing plants are situated in different parts of the world, including Italy, Brazil, the United States, China, and others. Indeed, various industries are in the business of bioplastic production, especially PHA thermoplastic material.

It has been evident that microalgae have proved to be a superlative candidate for the production of bioplastic but needs to be explored further in order to achieve commercial production of bioplastic emanated from microalgae independently or in consortium with bacteria. A substitute for a plastic bottle was also prepared by the composition of red algae powder and water (Verlinden et al., 2007; Singh et al., 2017; Alcântara et al., 2020; Chia et al., 2020; Price et al., 2020; Shafqat et al., 2020). Therefore, commercialization of bioplastic emanated from algae can be achieved by investigating the bioplastic production potential of various algal species along with the metabolic engineering and evaluation of multiple aspects influencing the accumulation of bioplastics. However, acquiring a more profound understanding of the know-how related to downstream processing as well as the participation of algae-derived plastics need to be the prime concern of the researchers, as they contribute considerably to the production cost of commercialized plastic.

## Concluding Remarks

Microbes are scrutinized substantially for the synthesis of sustainable bioplastics and had proven to be a magnificent source of bioplastic precursors that could be utilized further to produce biocompatible and carbon-neutral biodegradable products. As per literature, bacteria have been reported to accumulate about 80–90% of PHA when subjected to optimal conditions. Currently, bacteria are consistently exploited to commercialize biodegradable and sustainable bacterial bioplastic, and many industries are doing business out of it. Despite bioplastic production *via* bacteria being commercialized, a cost-effective, sustainable large-scale venture for the synthesis of bioplastic, especially PHA bioplastic, is still in its early years. The challenges faced in the process include the need for expensive carbon sources, operational cost to maintain the process control parameters, low yield of a metabolite of interest, difficulty in achieving considerable productivity, and downstream processing steps. Whereas, usage photosynthetic organisms are still in their early course nut they have immense leeway for achieving a greater place in bioplastic industries. From the past few decades, microalgae and cyanobacteria are also under surveillance for evaluating their potential of bioplastic precursor accumulation or for direct usage in bioplastic production. Nevertheless, the methodologies evolved for the production of algal bioplastic are not economically feasible and environmentally sustainable. Additionally, many technical and procedural complications occur while scaling up bioplastic production emanated from algae.

Keeping in mind the various pro and cons of PHA production *via* various microbes individually, a consortium approach seems to be a light in the dusk. In recent years, different MMCs are under observation to uncover their bioplastic production potential, either by tweaking certain culture conditions or by metabolic engineering approach. The practice of utilizing photosynthetic MMCs composed of bacteria and algae consortia or different photosynthetic bacteria, accompanied by the consumption of wastewater or byproducts of various production processes like molasses, grape pomace, olive pomace, and crude glycerol as a substitute for pure organic carbon source, will prove to be an efficacious perspective for the production of economically feasible, environmentally sustainable, and biodegradable bioplastic. PHA produced by MMCs can also have a greater composition for a better composition and mechanical properties.

A biorefinery approach can also help overcome the cost and sustainability crisis in commercialization of PHA as bioplastic. Tailor-made consortium of microalgae and photosynthetic bacteria will attain high productivity of PHA as well as precursors of bioenergy molecules to spread out the burden of scaling up. In a nutshell, promising biodegradable bioplastic constituents can be derived from algae and bacterial consortia as an alternative for replacing traditional fossil-based plastics, ultimately unraveling the strategy of reducing plastic pollution as well as the burden on the finite resources.

## Author Contributions

KS worked on conceptualization, methodology, and visualization. RS gave her contribution in data curation and writing the original draft. RN reviewed and edited the manuscript. KB supervised and conceived the manuscript. Manuscript was critically reviewed and approved by all the authors.

## Conflict of Interest

The authors declare that the research was conducted in the absence of any commercial or financial relationships that could be construed as a potential conflict of interest.

## Publisher’s Note

All claims expressed in this article are solely those of the authors and do not necessarily represent those of their affiliated organizations, or those of the publisher, the editors and the reviewers. Any product that may be evaluated in this article, or claim that may be made by its manufacturer, is not guaranteed or endorsed by the publisher.

## Abbreviations

CO_2_: Carbon dioxide
COD: Chemical oxygen demand
HRAP: High-rate algal ponds
Lcl-pha: Long-chain length polyhydroxyalkanoate
Mcl-pha: Medium-chain length polyhydroxyalkanoate
MMC: Mixed microbial culture
M_w_: Molecular weight
NADP: Nicotinamide adenine dinucleotide hydride
NADPH: Nicotinamide adenine dinucleotide phosphate hydride
PHA: Polyhydroxyalkanoate
PHB: Polyhydroxybutyrate
PHBH: Poly-(3-hydroxybutyrate-co-3-hydroxyhexanoate)
PHBV: Polyhydroxybutyrate-co-valerate
PMC: photosynthetic microbial culture
PNSB: Purple non sulfur bacteria
PSB: Purple sulfur bacteria
Scl-PHA: short-chain-length Polyhydroxyalkanoate
SUP: Single-use plastics
VFA: Volatile fatty acid
VSS: Volatile suspended solid
